# (*E*)-1-Methyl-5-(3-methyl-4-chloro­phen­oxy)-3-trifluoro­meth­yl-1*H*-pyrazole-4-carbaldehyde *O*-acetyl­oxime

**DOI:** 10.1107/S1600536811006696

**Published:** 2011-02-26

**Authors:** Hong Dai, Yan-Fei Shen, Jiao Chen, Hong-Lian Chen, Yong-Jun Shen

**Affiliations:** a College of Chemistry and Chemical Engineering, Nantong University, Nantong 226019, People’s Republic of China

## Abstract

In the title mol­ecule, C_15_H_13_ClF_3_N_3_O_3_, the pyrazole and benzene rings form a dihedral angle of 77.6 (3)°. In the crystal, mol­ecules related by translation along the *a* axis are linked into chains *via* C—H⋯O hydrogen bonds. The crystal packing is stabilized further by weak π–π [centroid–centroid distance = 3.734 (6) Å] and dipole–dipole inter­actions [C⋯O = 3.174 (2) Å].

## Related literature

For the bioactivity of pyrazole derivatives, see: Hagiwara & Suzuki (1996 ▶); Ranatunge *et al.* (2004 ▶). For related structures, see: Fu *et al.* (2008 ▶); Li *et al.* (2006 ▶). For the biological activity of compounds containing an oxime ester fragment, see: Vonhoff *et al.* (1999 ▶); Wood *et al.* (1997 ▶).
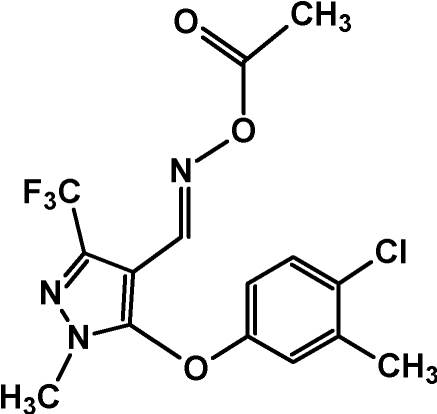


## Experimental

### 

#### Crystal data

C_15_H_13_ClF_3_N_3_O_3_
*M* *_r_* = 375.73
Orthorhombic, 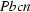

*a* = 11.951 (2) Å
*b* = 19.549 (4) Å
*c* = 13.726 (3) Å
*V* = 3206.8 (11) Å^3^
*Z* = 8
Mo *K*α radiation
μ = 0.29 mm^−1^
*T* = 113 K
0.16 × 0.12 × 0.08 mm

#### Data collection

Rigaku Saturn diffractometer
Absorption correction: multi-scan (*CrystalClear*; Rigaku, 2008 ▶) *T*_min_ = 0.955, *T*_max_ = 0.977
21575 measured reflections
3686 independent reflections
3208 reflections with *I* > 2σ(*I*)
*R*_int_ = 0.061

#### Refinement

*R*[*F* ^2^ > 2σ(*F* ^2^)] = 0.046
*wR*(*F* ^2^) = 0.115
*S* = 1.10
3686 reflections
229 parameters
H-atom parameters constrained
Δρ_max_ = 0.29 e Å^−3^
Δρ_min_ = −0.33 e Å^−3^

### 

Data collection: *CrystalClear* (Rigaku, 2008 ▶); cell refinement: *CrystalClear*; data reduction: *CrystalClear*; program(s) used to solve structure: *SHELXS97* (Sheldrick, 2008 ▶); program(s) used to refine structure: *SHELXL97* (Sheldrick, 2008 ▶); molecular graphics: *SHELXTL* (Sheldrick, 2008 ▶); software used to prepare material for publication: *SHELXTL*.

## Supplementary Material

Crystal structure: contains datablocks global, I. DOI: 10.1107/S1600536811006696/cv5055sup1.cif

Structure factors: contains datablocks I. DOI: 10.1107/S1600536811006696/cv5055Isup2.hkl

Additional supplementary materials:  crystallographic information; 3D view; checkCIF report

## Figures and Tables

**Table 1:** Hydrogen-bond geometry (Å, °)

*D*—H⋯*A*	*D*—H	H⋯*A*	*D*⋯*A*	*D*—H⋯*A*
C5—H5*B*⋯O3^i^	0.96	2.55	3.102 (2)	117

Symmetry code: (i) 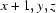.

**Fig. 1.**
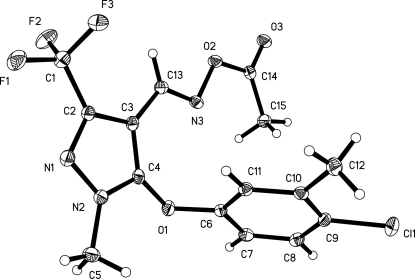
The molecular structure of (I) showing the atomic numbering and displacement ellipsoids drawn at the 30% probability level.

**Fig. 2.**
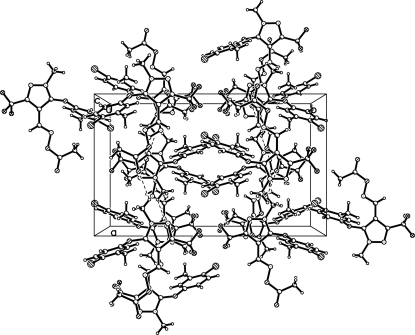
A portion of the crystal packing of (I). Hydrogen bonds drawn as dashed lines.

## supplementary crystallographic information

### Comment

Pyrazole derivatives have been paid much attention due to their diverse biological activities. Some of them are used as fungicide, insecticide, and antitumor agents (Hagiwara & Suzuki, 1996; Ranatunge *et al.*, 2004). Several studies have recently reported the crystal structures of related pyrazole compounds (Li *et al.*, 2006; Fu *et al.*, 2008). On the other hand, oxime ester group as an efficient pharmacophore was widely used in the field of agricultural and medicinal chemistry (Wood *et al.* , 1997; Vonhoff *et al.*, 1999). Motivated by the above observations and in continuation of research on the bioactivities of pyrazole derivatives, we synthesized the title compound (I).

In (I) (Fig. 1), the dihedral angle between the planes of the phenyl ring and the pyrazoe ring is 77.6 (3)°. In the crystal structure, the molecules related by translation along axis *a* are linked into chains *via* C—H···O hydrogen bonds (Table 2; Fig. 2). The crystal packing is stabilized further by the weak π—π and dipole-dipole interactions (Table 1).

### Experimental

To a stirred solution of 1-methyl-3-(trifluoromethyl)-5-phenoxy-1*H*- pyrazole-4-carbaldehyde oxime (8 mmol),and sodium bicarbonate (20 mmol) in 80 ml of chloroform, was added dropwise acetyl chloride(10 mmol) at room temperature. The reaction mixture was heated to reflux for 8 h. After cooling to room temperature, the mixture was washed with water (3 * 10 ml) and then with saturated brine (3 * 20 ml), and dried over anhydrous sodium sulfate. The solvent was evaporated under reduced pressure, and the residue was recrystallized from petroleum ether/ethyl acetate (8:1 *v*/*v*) to obtain colourless crystals.

### Refinement

All H atoms were placed in calculated positions, with C–H = 0.93 - 0.96 Å, and included in the final cycles of refinement using a riding model, with *U*_iso_(H) = 1.2-1.5 *U*_eq_(C).

### Crystal data

**Table:**

C_15_H_13_ClF_3_N_3_O_3_	*F*(000) = 1536
*M**_r_* = 375.73	*D*_x_ = 1.557 Mg m^−^^3^
Orthorhombic, *P**b**c**n*	Mo *K*α radiation, λ = 0.71073 Å
Hall symbol: -P 2n 2ab	Cell parameters from 7040 reflections
*a* = 11.951 (2) Å	θ = 2.0–27.5°
*b* = 19.549 (4) Å	µ = 0.29 mm^−^^1^
*c* = 13.726 (3) Å	*T* = 113 K
*V* = 3206.8 (11) Å^3^	Orthorhombic, colourless
*Z* = 8	0.16 × 0.12 × 0.08 mm

### Data collection

**Table:**

Rigaku Saturn diffractometer	3686 independent reflections
Radiation source: rotating anode	3208 reflections with *I* > 2σ(*I*)
confocal	*R*_int_ = 0.061
ω scans	θ_max_ = 27.5°, θ_min_ = 2.0°
Absorption correction: multi-scan (*CrystalClear*; Rigaku, 2008)	*h* = −12→15
*T*_min_ = 0.955, *T*_max_ = 0.977	*k* = −25→24
21575 measured reflections	*l* = −14→17

### Refinement

**Table:**

Refinement on *F*^2^	Primary atom site location: structure-invariant direct methods
Least-squares matrix: full	Secondary atom site location: difference Fourier map
*R*[*F*^2^ > 2σ(*F*^2^)] = 0.046	Hydrogen site location: inferred from neighbouring sites
*wR*(*F*^2^) = 0.115	H-atom parameters constrained
*S* = 1.10	*w* = 1/[σ^2^(*F*_o_^2^) + (0.0516*P*)^2^ + 1.0705*P*] where *P* = (*F*_o_^2^ + 2*F*_c_^2^)/3
3686 reflections	(Δ/σ)_max_ = 0.005
229 parameters	Δρ_max_ = 0.29 e Å^−^^3^
0 restraints	Δρ_min_ = −0.33 e Å^−^^3^

### Special details

**Table:**

Geometry. All e.s.d.'s (except the e.s.d. in the dihedral angle between two l.s. planes) are estimated using the full covariance matrix. The cell e.s.d.'s are taken into account individually in the estimation of e.s.d.'s in distances, angles and torsion angles; correlations between e.s.d.'s in cell parameters are only used when they are defined by crystal symmetry. An approximate (isotropic) treatment of cell e.s.d.'s is used for estimating e.s.d.'s involving l.s. planes.
Refinement. Refinement of *F*^2^ against ALL reflections. The weighted *R*-factor *wR* and goodness of fit *S* are based on *F*^2^, conventional *R*-factors *R* are based on *F*, with *F* set to zero for negative *F*^2^. The threshold expression of *F*^2^ > σ(*F*^2^) is used only for calculating *R*-factors(gt) *etc*. and is not relevant to the choice of reflections for refinement. *R*-factors based on *F*^2^ are statistically about twice as large as those based on *F*, and *R*- factors based on ALL data will be even larger.

### Fractional atomic coordinates and isotropic or equivalent isotropic displacement parameters (Å^2^)

**Table:**

	*x*	*y*	*z*	*U*_iso_*/*U*_eq_	
Cl1	0.78669 (4)	1.02584 (3)	1.15028 (4)	0.03467 (15)	
F1	1.04265 (9)	0.54780 (6)	0.90733 (11)	0.0422 (3)	
F2	0.89699 (11)	0.56763 (6)	0.82078 (10)	0.0429 (3)	
F3	0.88936 (10)	0.57911 (6)	0.97538 (10)	0.0415 (3)	
O1	0.97587 (11)	0.84054 (6)	0.85735 (9)	0.0258 (3)	
O2	0.62455 (10)	0.75817 (7)	0.87263 (9)	0.0254 (3)	
O3	0.45722 (10)	0.80203 (7)	0.86552 (10)	0.0311 (3)	
N1	1.09611 (12)	0.67936 (8)	0.88946 (11)	0.0254 (3)	
N2	1.09519 (12)	0.74822 (8)	0.88089 (11)	0.0236 (3)	
N3	0.74122 (12)	0.77434 (8)	0.87076 (11)	0.0233 (3)	
C1	0.95593 (15)	0.58904 (10)	0.89804 (14)	0.0282 (4)	
C2	0.98845 (14)	0.66220 (9)	0.88872 (13)	0.0221 (4)	
C3	0.91641 (14)	0.71893 (9)	0.87938 (12)	0.0216 (4)	
C4	0.99049 (14)	0.77333 (9)	0.87439 (12)	0.0214 (4)	
C5	1.19918 (15)	0.78688 (11)	0.87754 (15)	0.0302 (4)	
H5A	1.2122	0.8025	0.8122	0.045*	
H5B	1.2600	0.7581	0.8978	0.045*	
H5C	1.1939	0.8255	0.9204	0.045*	
C6	0.93017 (13)	0.88174 (9)	0.93094 (12)	0.0204 (3)	
C7	0.89984 (14)	0.94648 (9)	0.90188 (13)	0.0240 (4)	
H7	0.9087	0.9602	0.8375	0.029*	
C8	0.85584 (14)	0.99074 (9)	0.97051 (14)	0.0252 (4)	
H8	0.8353	1.0349	0.9529	0.030*	
C9	0.84251 (14)	0.96877 (9)	1.06574 (13)	0.0231 (4)	
C10	0.87284 (13)	0.90360 (9)	1.09597 (13)	0.0217 (4)	
C11	0.91814 (13)	0.85973 (9)	1.02608 (13)	0.0215 (4)	
H11	0.9402	0.8158	1.0435	0.026*	
C12	0.85611 (16)	0.87976 (10)	1.19861 (14)	0.0293 (4)	
H12A	0.8918	0.9111	1.2426	0.044*	
H12B	0.8882	0.8351	1.2063	0.044*	
H12C	0.7775	0.8778	1.2128	0.044*	
C13	0.79576 (14)	0.71860 (9)	0.87804 (13)	0.0227 (4)	
H13	0.7574	0.6773	0.8825	0.027*	
C14	0.55571 (14)	0.81436 (10)	0.86479 (13)	0.0246 (4)	
C15	0.60736 (15)	0.88311 (10)	0.85610 (16)	0.0309 (4)	
H15A	0.5496	0.9172	0.8527	0.046*	
H15B	0.6522	0.8850	0.7981	0.046*	
H15C	0.6537	0.8916	0.9119	0.046*	

### Atomic displacement parameters (Å^2^)

**Table:**

	*U*^11^	*U*^22^	*U*^33^	*U*^12^	*U*^13^	*U*^23^
Cl1	0.0395 (3)	0.0301 (3)	0.0343 (3)	0.00681 (19)	−0.00035 (19)	−0.0103 (2)
F1	0.0363 (6)	0.0256 (6)	0.0646 (9)	0.0072 (5)	−0.0057 (6)	0.0028 (6)
F2	0.0556 (8)	0.0291 (7)	0.0440 (7)	−0.0061 (5)	−0.0190 (6)	−0.0062 (6)
F3	0.0475 (7)	0.0326 (7)	0.0446 (8)	−0.0076 (5)	0.0115 (6)	0.0049 (6)
O1	0.0355 (7)	0.0212 (7)	0.0206 (6)	0.0024 (5)	0.0072 (5)	0.0023 (5)
O2	0.0190 (6)	0.0244 (7)	0.0328 (7)	−0.0022 (5)	0.0000 (5)	0.0013 (6)
O3	0.0209 (6)	0.0340 (8)	0.0382 (8)	−0.0022 (5)	0.0028 (5)	0.0006 (6)
N1	0.0260 (8)	0.0245 (8)	0.0259 (8)	0.0014 (6)	−0.0005 (6)	−0.0024 (6)
N2	0.0237 (7)	0.0239 (8)	0.0232 (8)	−0.0007 (6)	0.0008 (6)	−0.0026 (6)
N3	0.0172 (7)	0.0284 (8)	0.0243 (8)	−0.0022 (6)	−0.0002 (6)	0.0002 (6)
C1	0.0285 (9)	0.0261 (10)	0.0300 (10)	0.0009 (7)	−0.0038 (8)	−0.0008 (8)
C2	0.0228 (8)	0.0224 (9)	0.0211 (9)	0.0010 (7)	−0.0004 (7)	−0.0019 (7)
C3	0.0230 (8)	0.0234 (9)	0.0183 (8)	0.0011 (7)	−0.0002 (6)	−0.0025 (7)
C4	0.0255 (8)	0.0230 (9)	0.0158 (8)	0.0026 (7)	0.0017 (6)	−0.0015 (7)
C5	0.0261 (9)	0.0350 (11)	0.0295 (10)	−0.0078 (8)	0.0024 (7)	−0.0038 (8)
C6	0.0194 (7)	0.0210 (9)	0.0208 (8)	−0.0014 (6)	0.0006 (6)	−0.0018 (7)
C7	0.0242 (8)	0.0247 (9)	0.0231 (9)	−0.0015 (7)	−0.0013 (7)	0.0052 (7)
C8	0.0240 (8)	0.0195 (9)	0.0321 (10)	0.0019 (7)	−0.0046 (7)	0.0011 (8)
C9	0.0207 (8)	0.0228 (9)	0.0258 (9)	−0.0004 (6)	−0.0006 (7)	−0.0054 (7)
C10	0.0183 (7)	0.0249 (9)	0.0219 (9)	−0.0030 (6)	−0.0015 (6)	−0.0003 (7)
C11	0.0216 (8)	0.0198 (8)	0.0230 (9)	−0.0002 (7)	−0.0004 (7)	0.0007 (7)
C12	0.0318 (9)	0.0332 (11)	0.0230 (9)	0.0005 (8)	0.0019 (7)	−0.0009 (8)
C13	0.0244 (8)	0.0231 (9)	0.0206 (8)	−0.0027 (7)	−0.0002 (6)	0.0000 (7)
C14	0.0237 (9)	0.0285 (10)	0.0216 (9)	0.0012 (7)	0.0019 (7)	−0.0009 (7)
C15	0.0253 (9)	0.0240 (10)	0.0436 (12)	0.0007 (7)	0.0045 (8)	−0.0007 (9)

### Geometric parameters (Å, °)

**Table:**

Cl1—C9	1.7425 (18)	C5—H5C	0.9600
F1—C1	1.319 (2)	C6—C7	1.376 (2)
F2—C1	1.340 (2)	C6—C11	1.382 (2)
F3—C1	1.341 (2)	C7—C8	1.383 (3)
O1—C4	1.346 (2)	C7—H7	0.9300
O1—C6	1.403 (2)	C8—C9	1.385 (3)
O2—C14	1.377 (2)	C8—H8	0.9300
O2—N3	1.4299 (18)	C9—C10	1.388 (3)
O3—C14	1.201 (2)	C10—C11	1.396 (2)
N1—C2	1.330 (2)	C10—C12	1.497 (2)
N1—N2	1.351 (2)	C11—H11	0.9300
N2—C4	1.347 (2)	C12—H12A	0.9600
N2—C5	1.455 (2)	C12—H12B	0.9600
N3—C13	1.274 (2)	C12—H12C	0.9600
C1—C2	1.488 (3)	C13—H13	0.9300
C2—C3	1.410 (2)	C14—C15	1.484 (3)
C3—C4	1.385 (3)	C15—H15A	0.9600
C3—C13	1.442 (2)	C15—H15B	0.9600
C5—H5A	0.9600	C15—H15C	0.9600
C5—H5B	0.9600		
			
C14···O3^i^	3.174 (2)	Cg···Cg^ii^	3.734 (6)
			
C4—O1—C6	119.07 (13)	C6—C7—H7	120.7
C14—O2—N3	113.89 (13)	C8—C7—H7	120.7
C2—N1—N2	104.05 (14)	C7—C8—C9	119.51 (17)
C4—N2—N1	112.11 (14)	C7—C8—H8	120.2
C4—N2—C5	127.00 (16)	C9—C8—H8	120.2
N1—N2—C5	120.88 (15)	C8—C9—C10	122.46 (17)
C13—N3—O2	107.97 (14)	C8—C9—Cl1	118.29 (14)
F1—C1—F2	107.37 (16)	C10—C9—Cl1	119.25 (14)
F1—C1—F3	107.53 (16)	C9—C10—C11	117.37 (16)
F2—C1—F3	105.63 (15)	C9—C10—C12	122.14 (16)
F1—C1—C2	112.99 (15)	C11—C10—C12	120.48 (16)
F2—C1—C2	111.69 (16)	C6—C11—C10	119.89 (16)
F3—C1—C2	111.24 (16)	C6—C11—H11	120.1
N1—C2—C3	113.16 (16)	C10—C11—H11	120.1
N1—C2—C1	119.65 (15)	C10—C12—H12A	109.5
C3—C2—C1	127.19 (16)	C10—C12—H12B	109.5
C4—C3—C2	102.59 (15)	H12A—C12—H12B	109.5
C4—C3—C13	129.93 (17)	C10—C12—H12C	109.5
C2—C3—C13	127.47 (17)	H12A—C12—H12C	109.5
O1—C4—N2	119.20 (16)	H12B—C12—H12C	109.5
O1—C4—C3	132.45 (16)	N3—C13—C3	120.59 (17)
N2—C4—C3	108.10 (16)	N3—C13—H13	119.7
N2—C5—H5A	109.5	C3—C13—H13	119.7
N2—C5—H5B	109.5	O3—C14—O2	115.13 (17)
H5A—C5—H5B	109.5	O3—C14—C15	126.16 (18)
N2—C5—H5C	109.5	O2—C14—C15	118.71 (15)
H5A—C5—H5C	109.5	C14—C15—H15A	109.5
H5B—C5—H5C	109.5	C14—C15—H15B	109.5
C7—C6—C11	122.19 (16)	H15A—C15—H15B	109.5
C7—C6—O1	114.96 (15)	C14—C15—H15C	109.5
C11—C6—O1	122.83 (15)	H15A—C15—H15C	109.5
C6—C7—C8	118.57 (17)	H15B—C15—H15C	109.5
			
C2—N1—N2—C4	−0.38 (19)	C2—C3—C4—N2	−0.22 (18)
C2—N1—N2—C5	−179.17 (16)	C13—C3—C4—N2	178.40 (17)
C14—O2—N3—C13	−179.70 (14)	C4—O1—C6—C7	−168.95 (15)
N2—N1—C2—C3	0.2 (2)	C4—O1—C6—C11	12.2 (2)
N2—N1—C2—C1	−179.32 (16)	C11—C6—C7—C8	0.0 (3)
F1—C1—C2—N1	0.6 (2)	O1—C6—C7—C8	−178.92 (15)
F2—C1—C2—N1	−120.56 (18)	C6—C7—C8—C9	−0.6 (3)
F3—C1—C2—N1	121.68 (18)	C7—C8—C9—C10	0.7 (3)
F1—C1—C2—C3	−178.87 (17)	C7—C8—C9—Cl1	−179.94 (13)
F2—C1—C2—C3	60.0 (2)	C8—C9—C10—C11	−0.1 (3)
F3—C1—C2—C3	−57.8 (2)	Cl1—C9—C10—C11	−179.43 (12)
N1—C2—C3—C4	0.0 (2)	C8—C9—C10—C12	−178.78 (16)
C1—C2—C3—C4	179.51 (17)	Cl1—C9—C10—C12	1.8 (2)
N1—C2—C3—C13	−178.68 (17)	C7—C6—C11—C10	0.7 (3)
C1—C2—C3—C13	0.8 (3)	O1—C6—C11—C10	179.45 (15)
C6—O1—C4—N2	−112.02 (17)	C9—C10—C11—C6	−0.6 (2)
C6—O1—C4—C3	74.6 (2)	C12—C10—C11—C6	178.15 (16)
N1—N2—C4—O1	−174.50 (14)	O2—N3—C13—C3	−179.26 (15)
C5—N2—C4—O1	4.2 (3)	C4—C3—C13—N3	0.5 (3)
N1—N2—C4—C3	0.4 (2)	C2—C3—C13—N3	178.78 (17)
C5—N2—C4—C3	179.09 (17)	N3—O2—C14—O3	179.52 (15)
C2—C3—C4—O1	173.74 (18)	N3—O2—C14—C15	−0.3 (2)
C13—C3—C4—O1	−7.6 (3)		

Symmetry codes: (i) −*x*+1, *y*, −*z*+3/2; (ii) −*x*+2, *y*, −*z*+3/2.

### Hydrogen-bond geometry (Å, °)

**Table:**

*D*—H···*A*	*D*—H	H···*A*	*D*···*A*	*D*—H···*A*
C5—H5B···O3^iii^	0.96	2.55	3.102 (2)	117

Symmetry codes: (iii) *x*+1, *y*, *z*.
